# Evidence for litter differences in play behaviour in pre-weaned pigs

**DOI:** 10.1016/j.applanim.2015.09.007

**Published:** 2015-11

**Authors:** Sarah Mills Brown, Michael Klaffenböck, Ian Macleod Nevison, Alistair Burnett Lawrence

**Affiliations:** a Division of Neurobiology, The Roslin Institute, The University of Edinburgh, Easter Bush, Midlothian EH25 9RG, United Kingdom; b University of Natural Resources and Life Sciences, Gregor-Mendel-Straβe 33, 1180 Vienna, Austria; c Biomathematics and Statistics Scotland, James Clerk Maxwell Building, Peter Guthrie Tait Road, Edinburgh EH9 3FD, United Kingdom; d Animal & Veterinary Sciences, SRUC, West Mains Road, Edinburgh EH9 3JG, United Kingdom

**Keywords:** Pig, Spontaneous play behaviour, Individual differences, Litter differences, Pre-natal, Post-natal, Growth development, Sex effects

## Abstract

The aim of this study was to analyse spontaneous play behaviour in litters of domestic pigs (*Sus scrofa*) for sources of variation at individual and litter levels and to relate variation in play to measures of pre and postnatal development. Seven litters of commercially bred piglets (*n* = 70) were born (farrowed) within a penning system (PigSAFE) that provided opportunities for the performance of spontaneous play behaviours. Individual behaviour was scored based on an established play ethogram for 2 days per week over the 3 week study period. We found strong evidence of litter differences in play behaviour (*F*_(6,63)_ = 27.30, *p* < 0.001). Of the variance in total play, 50% was attributable to differences between litters with a lesser proportion (11%) to between piglets within litters. We found similar evidence of litter differences when we analysed the separate play categories (e.g. for locomotor play: *F*_(6,63)_ = 27.50, *p* < 0.001). For social and locomotor play the variance was partitioned in a broadly similar way to total play; however for object play the variance was distributed with a more even balance across and within litters. In terms of explanatory factors we found little evidence that at the litter level differences in play were associated with differences in general activity. Of the prenatal factors measured, we found that birth weight was positively associated with total play and the play categories (e.g. with total play: *F*_(1,64)_ = 12.8, *p* < 0.001). We also found that postnatal piglet growth up to weaning (as a percentage of birth weight) had a significant positive association with total play and the play categories (e.g. with object play: *F*_(1,66)_ = 20.55, *p* < 0.001). As found in other studies, on average males engaged in more social play (e.g. non-injurious play fighting: *F*_(1,63)_ = 39.8, *p* < 0.001). Males also initiated more play bouts on average than females (*F*_(1,62)_ = 4.41, *p* = 0.040). We conclude that the study of differences between litters and individuals provides a robust approach to understanding factors potentially influencing play behaviour in the pig. This work also provides support for the use of play as a welfare indicator in pre-weaned piglets as the litter differences in play we observed were associated positively with physical development.

## Highlights

• First demonstration of between litter differences in play behaviour in pigs.
• Litter differences in play behaviour appear independent of overall activity levels.
• Litter differences in play behaviour associate strongly with post-natal growth.
• Pre-natal factors (particularly birth weight and BMI) associate positively with play behaviour.
• Pre-weaning play behaviour has potential as an indicator of positive welfare.

## 1 Introduction

Play is a commonly observed and characteristic behaviour of young mammals (e.g. Bekoff and Byers, 1998). Despite difficulties posed by the scientific study of play behaviour (e.g. Burghardt, 2005) it has been and remains a topic of considerable interest in the behavioural sciences (see Graham and Burghardt, 2010, Held and Spinka, 2011 for recent reviews). Recent studies have aimed to understand the function of play (e.g. Cameron et al., 2008), and the mechanisms underlying play behaviour including analyses of the neural networks associated with and potentially causal to play behaviour (e.g. Northcutt and Nguyen, 2014). Play also has applied relevance as it has been suggested as a potential indicator of high levels of animal welfare given that play tends to be expressed only under good or ‘optimal’ environmental conditions (e.g. Lawrence, 1987, Held and Spinka, 2011).

Pig play behaviour has been described in wild and domesticated species (e.g. Frädich, 1974, Dobao et al., 1985), and generally has similarities to play found in other species of young mammal. For example play in pigs is age-dependant. In a study of play in domesticated pigs (*Sus scrofa*) living in a semi-natural environment (Newberry et al., 1988), play increased in the first 6 weeks of life but thereafter declined to low levels by week 14 of life. As with other species, play behaviour in pigs can be categorised into locomotor, object-directed and social play (e.g. Blackshaw et al., 1997). The behaviours that are recognised as play in pigs have some resemblance to adult behaviours (e.g. running; play fighting) but at the same time are recognisably different, being performed in an exaggerated, energetic and repetitive manner (e.g. Newberry et al., 1988).

The study of individual differences in behaviour has become of considerable interest in behavioural science and there is a growing body of literature (reviewed by Bell et al., 2009) reporting that individuals across different species show consistent differences in behaviour (e.g. aggression (Dingemanse et al., 2007), exploratory behaviour (Quinn and Cresswell, 2005)). Individual behavioural differences provide one approach to the study of behavioural genetics (e.g. Turner et al., 2008), to the proximate mechanisms underlying behaviours (e.g. Andari et al., 2014) and to the function of behaviour (e.g. Laskowski and Bell, 2014). Despite the general interest in individual differences in behaviour, there are few studies that set out to specifically look for stable individual differences in play behaviour (see Held and Spinka, 2011). In polytocous species there have been only a few studies studying the consistency of play within and across litters with reports of consistent litter differences in play in cats (Martin and Bateson, 1985) and dogs (Pal, 2010). There have been no similar studies in the pig although a recent study (Rauw, 2013) found that litter of origin was significantly associated with play behaviour in post-weaned pigs.

The aim of this study was to analyse spontaneous play behaviour in pre-weaned pigs for evidence of litter and individual differences in play behaviour and also to estimate the proportional distribution of variation in play behaviour between its different constituents. The pigs were born and reared in an environment that provided opportunities (space and ‘enrichment’) for the performance of play behaviours. We additionally collected other data on the piglets relating to their pre and post-natal development in order to investigate associations of potential explanatory variables with observed within and between litter differences in play behaviour.

## 2 Material and methods

### 2.1 Animals and housing

The 70 piglets that were studied were bred from seven commercial cross-bred dams (Large White × Landrace); the boar-line was American Hampshire. Litters were born within a 4 day time window. Litter size was not standardised and was dependent on biological variation (9–12 piglets per litter in this study). Cross fostering was kept to a minimum and only performed where piglet welfare was considered at risk.

**Fig. 1:**
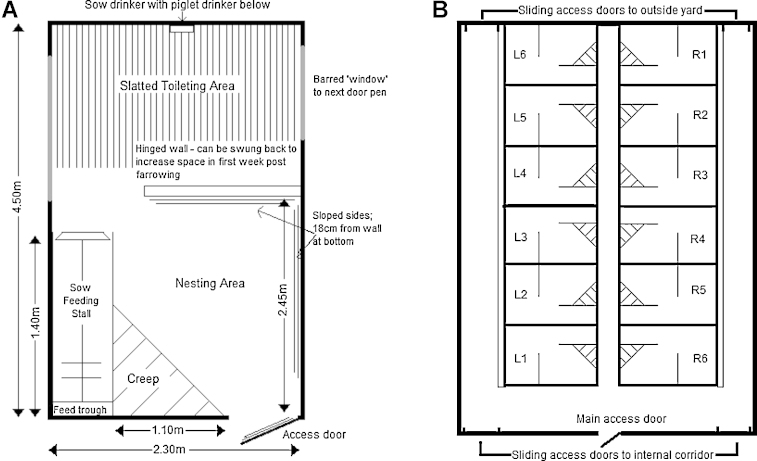
Diagram of PigSAFE pen (A) and building layout (B). Piglets and sows used in this study were housed in pens L1, L2, L3, R2, R4, R5 and R6. There were also litters in pens L4, R3 and R1 which were not part of the study.

The experimental animals were housed in the *Pig and Sow Alternative Farrowing Environment* (PigSAFE) pens (Edwards et al., 2012). PigSAFE pens allow species-specific behaviours in both the sow and the piglets to be expressed by providing more space and the possibility for provision of straw (1 kg per pen per day, approximately) as a substrate for ‘environmental enrichment’ compared to conventional farrowing environments (Fig. 1). No other manipulable materials were provided. Temperature within the unit was controlled in accordance to the Defra Code of Recommendations for the Welfare of Livestock (Defra, 2003), and pigs were maintained on a 12 h light/dark cycle. Piglets were managed according to standard farm practice (UK) including iron injection at 3 days of age, vaccination against Porcine Circoviral Disease (PCVD) at 28 days of age and ear tagging for identification at weaning. No tooth clipping was performed and males were not castrated.

### 2.2 Piglet Measures

Within 24 h of birth piglets were measured manually from crown of the head to base of tail (as reported in Baxter et al., 2008) to within 5 mm. Piglets were also weighed at this stage and at weekly intervals (based on birth date) up to weaning. We estimated ponderal index (PI = weight (kg)/length (m)^3^) and body mass index (BMI = weight (kg)/length (m)^2^) which have both been shown to be relevant indicators of pre-natal development in the pig (e.g. Baxter et al., 2008). Litter size was the number of piglets that survived beyond the first 2 weeks post farrowing. Post-natal growth was calculated as the percentage change in mass from birth to weaning.

### 2.3 Ethical approval

This project was reviewed and approved by SRUC (Scotland's Rural College) ethical review committee. All routine animal management procedures were adhered to by trained staff and health issues treated as required. All piglets were returned to commercial stock at the end of the study.

### 2.4 Experimental Design

The experiment spanned approximately 27 days from farrowing until weaning. Play behaviours were determined largely using an ethogram based on previous work in pigs (see Table 1); non-harmful fighting was included in the category of social play.

### 2.5 Recording of play behaviours

The animals were digitally recorded from day 1 using Sony LL20 low light cameras with infra-red and a Geovision GV-DVR. Two cameras were set up per pen, one at the rear and one at the front to provide maximal coverage. Piglets were not visible when in the creep box but could be seen at all other times. Behavioural observations were started when piglets were approximately 1 week old and continued with two observations per week (Mondays and Fridays from 0900 until 1300) until the piglets were weaned (six observation days in total).

On observation days (between 0800 and 0900), piglets were numbered on the back with numbers corresponding to their post-farrowing ID's using a black permanent marker. Cameras were set to record and video data analysed for the time period 0900–1300. The time period was chosen to commence after early morning husbandry and to extend for a period that would contain sufficient play bouts for analysis. The collected video material was searched for play bouts, defined as episodes where at least one piglet was observed to engage in playful behaviour. Play behaviour for each individual piglet during these play bouts was then recorded using focal sampling with Noldus’ *The Observer XT 11* (Noldus Information Technology bv, Wageningen, the Netherlands) software package. A coding scheme was created, relating each behaviour from the ethogram and every individual piglet with a specific key. Where more than one animal were observed starting a play bout simultaneously, the video was analysed for one animal and then rewound and analysed for the others. All data recorded was in the form of frequency counts. One observer completed all video analysis to remove any reliability issues relating to multiple observers.

### 2.6 Activity score

On observation days, an activity score for each individual piglet was recorded on an Excel spread sheet during a 5 s window every half hour between 0900 and 1300. Individuals were defined as active when they were moving around the pen or lying/sitting but showing movement of the body and/or head. Individuals were inactive when lying with no movement or out of site in the creep area. The activity score was calculated as the sum of all times active during the observational period resulting in an individual activity score for each experimental animal per observation day.

### 2.7 Statistical analysis

Basic descriptive statistics were calculated using Minitab 16. All further analysis was carried out using Genstat (16th Edition). In order to more closely satisfy the assumptions underlying the statistical methods applied, count data were square root transformed and percentage data were arcsine transformed. The activity score did not require transformation.

We addressed the statistical analysis of within and between litter differences in play in two ways. The first of these treated litter as a fixed effect, as did Martin and Bateson (1985). We formally compared litters for differences in square root transformed counts of total play, the different play categories (locomotor, object and social) and the different play elements (see Table 1), and activity. We used one-way Analysis of Variance to compare litters with one value per individual (being the average of the transformed values from each of the six observations days). The second approach was to fit a mixed model (i.e. a model comprising both fixed and random effects) in the GenStat statistical package using the REML algorithm. This approach broadens the inference from the specific litters studied to the population of litters. The random effects part of the model comprised four terms: litter, litter × observation day, piglet within litter and residual variation providing estimates of variance components for these four sources of variation. Thus the variance component for litter is an estimate of the variance in the population of litters from which the seven observed in this study were a sample. The fixed effects part of the model comprised observation day and sex. This provided a formal statistical test for sex differences. From the estimated variance components the variance for the mean for a single observed animal was calculated together with the percentage contribution of each of the four sources of variation to that variance. The potential for correlation between observations on different measurement days was modelled using a compound symmetry formulation; i.e. a common correlation for the residual variation between observation days was assumed. More complex correlation structures were not found to be useful based on a comparison of deviances.

Potential associations with prenatal and postnatal factors were explored through a stepwise fixed effects selection process within a mixed model framework (REML) applied to piglet means of the transformed behaviour data. The base model comprised litter as a random effect and no fixed effects. Other covariates (such as birth weight, BMI, sex, etc.) were added sequentially to the fixed effects model in the order of greatest statistical significance until no further terms gave a significant improvement. Pearson's product moment correlations were used to determine associations between measures at the between-litter level.

In the fixed effects model testing for litter differences by one-way Analysis of Variance the residual degrees of freedom was 63 after estimating a parameter for each litter. There was a slight imbalance between litters in the sex ratio and also the values of the various covariates (e.g. piglet birth weight) varied both between and within litters. Hence at both these levels there was information from which effects could be estimated. The REML analysis combined the between-litter and within-litter estimates of effects to give a single estimate. However, the relative prominence given to the two constituent estimates in the combined value depends on the relative precisions of the constituent estimates and this is also reflected in the residual degrees of freedom.

## 3 Results

### 3.1 Total Play Behaviour

**Fig. 2:**
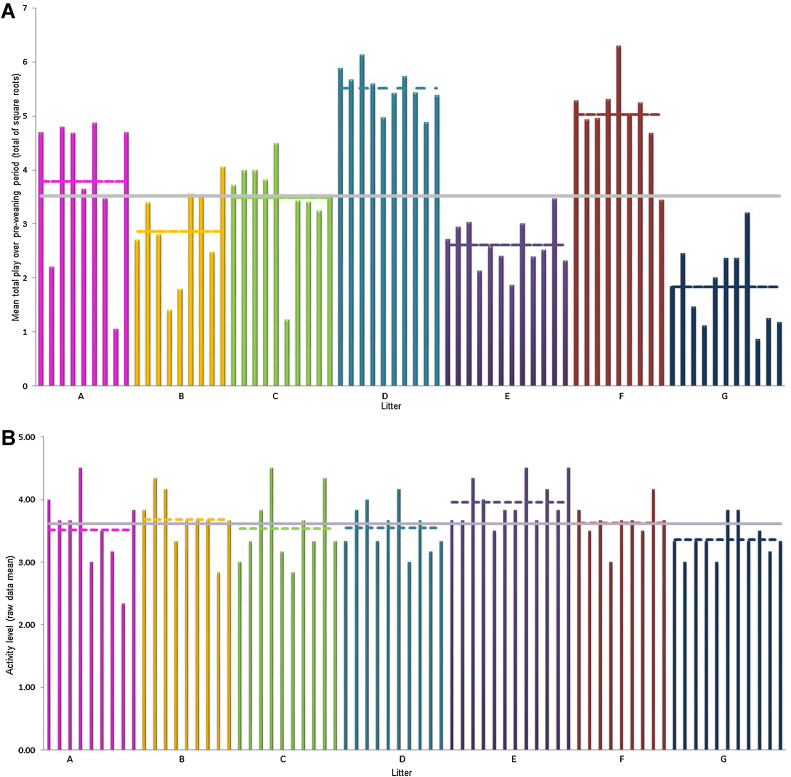
Graphical representation of total play counts (A) and activity (scored separately to play) (B) of each piglet in each litter averaged over the six observational days. In line with analysis mean total play is displayed as total of square root transformed and average activity counts as raw data. Horizontal coloured lines are the mean values for that litter while the horizontal grey lines represent the overall mean. Litters are labelled as A–G on the *x*-axis.

Analysing litter as a fixed effect, we found mean total play (counts) differed significantly between litters (*F*_(6,63)_ = 27.30, *p* < 0.001) (Fig. 2A). There was weak statistical evidence for litter differences in mean overall activity levels during the pre- weaning period (*F*_(6,63)_ = 2.15, *p* = 0.060) (Fig. 2B).

When we used REML to analyse the variance components for total play we estimated that for total play (averaged over observation days for a randomly selected pig of any given sex) 50% of the variance originated at the litter level, with 24% from a litter × observation day interaction and 11% from differences between piglets within litters (see Table 2). The REML analysis therefore suggests there is both between and within litter variation in total play with between litter variation being much the stronger effect. The REML analysis also showed that males displayed marginally higher mean levels of total play than females (total play counts (transformed): Males: 3.77 vs. Females: 3.36, SED = 0.20, *F*_(1,62)_ = 4.41, *p* = 0.04). Estimation of the variance components for general activity using REML showed 76% of the variation was due to residual variation (Table 2; Fig. 2B).

### 3.2 Play categories

On average, based on counts piglet play was 43% locomotor, 20.3% object and 36.7% social. Analysing with litter as a fixed effect we found strong evidence of litter differences in the mean absolute levels of all three play categories (Locomotor *F*_(6,63)_ = 27.50, *p* < 0.001; Object *F*_(6,63)_ = 10.94, *p* < 0.001; Social *F*_(6,63)_ = 12.94, *p* < 0.001). Observed differences between litters in the percentage of play in the different play categories did not reach significance (Locomotor *F*_(6,63)_ = 2.24, *p* = 0.051; Object *F*_(6,63)_ = 0.26, *p* = 0.955; Social *F*_(6,63)_ = 2.21, *p* = 0.053).

Using REML to estimate variance components we found, as with total play, evidence of between and within litter differences in the absolute levels of the play categories. For locomotor and social play the variance was partitioned in a broadly similar way to total play (Table 2); however for object play the variance was distributed somewhat differently with a more even balance across and within litters.

The REML analysis also found that males engaged in more total social play behaviours than females (counts for mean social play (transformed): Males = 2.447 vs. Females = 1.704, SED = 0.142, *F*_(1,63)_ = 27.3, *p* < 0.001). Neither locomotor nor object play showed any evidence for sex differences in absolute values. Piglets also displayed sex differences in the percentage of the type of play behaviour they performed, with females engaging on a percentage basis in more locomotor play behaviours (mean percentage of locomotor play (transformed): Females = 43.88 vs. Male mean = 37.65, SED = 2.182, *F*_(1,67)_ = 8.2, *p* = 0.006) while males engaged in more social play behaviours (mean percentage of social play (transformed): Females = 31.88 vs. Males mean = 41.16, SED = 1.471, *F*_(1,66)_ = 39.8, *p* < 0.001).

### 3.3 Play elements

We found that the sex differences in absolute levels of social play could be attributed to higher levels of non-harmful fighting in males (e.g. using REML: mean counts of non-harmful fighting (transformed): Males = 1.74 vs. Females = 1.04, SED 0.11, *F*_(1,63)_ = 39.8, *p* < 0.001) and pushing (mean counts of pushing (transformed): Males = 1.09 vs. Females = 0.76, SED = 0.10, *F*_(1,63)_ = 11.8, *p* < 0.001). We found similar effects for percentages of non-harmful fighting elements (e.g. mean percentage of counts of pushing (transformed): Males = 18.24 vs. Females = 14.24, SED = 1.41, *p* = 0.006).

REML analysis also indicated that the different play elements showed differences relative to each other in their partitioning of variance across the components (Table 3). For example, some elements (e.g. nudge and run) showed a similar distribution across the components to the play categories and total play, whilst others (e.g. hop and pivot) showed higher residual variation.

At the litter level the percentage of the elements ‘run’ and ‘flop’ were positively correlated with overall absolute total play in the pre-weaning period (correlations with total play using litter means of totals of square roots (*n* = 7): Run: *r* = 0.79, *p* = 0.033; Flop: *r* = 0.96, *p* < 0.001). No strong correlations were found between total play and other behavioural elements.

**Fig. 3:**
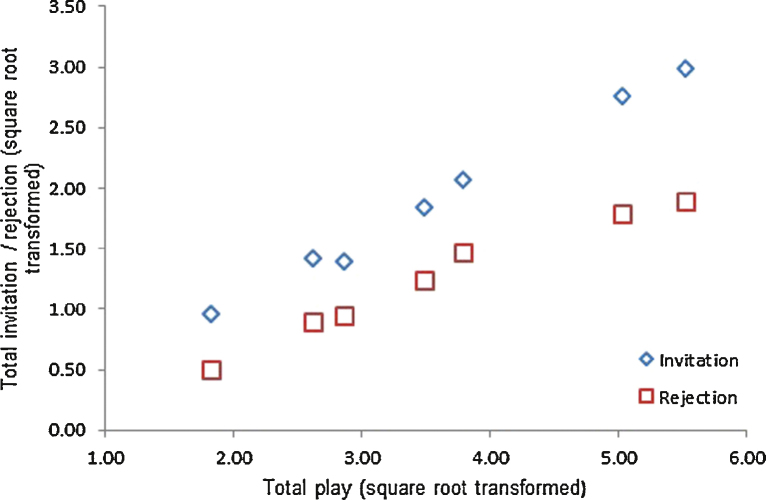
Litter means averaged over observation periods for invitation and rejections (square root transformed counts) plotted against total play across litters. Invitations are denoted by diamonds, rejections by squares.

Play invitations and rejections were considered separately from social play (following Martin et al., 2015). Using litter as a fixed factor there was a statistically significant difference in the mean play invitations and rejections across litters (Mean invitations *F*_(6,63)_ = 10.89, *p* < 0.001; Mean rejections *F*_(6,63)_ = 23.72, *p* < 0.001) which correlated strongly with total play levels (correlations with total play using litter means (*n* = 7): Invitations *r* = 0.858, *p* = 0.014; Rejections *r* = 0.766, *p* = 0.045; Fig. 3). There was no statistical evidence that the average ratio of play invitations to rejections differed across litters (*F*_(6,63)_ = 1.42, *p* = 0.22). Overall males initiated more play bouts per observation day than females (mean play initiations (transformed): Males: 2.24 vs. Females = 1.55; SED = 0.16; *F*_(1,63)_ = 19.22, *p* < 0.001).

Estimation of variance components using REML indicated that invitations and rejections showed a similar distribution of variance across components to total play and the play categories (e.g. 42% of variance in invitations and 40% in rejections was at the litter level).

### 3.4 Covariate analyses

Of the prenatal measures we found that birth weight was positively associated with total play (*F*_(1,64)_ = 12.8, *p* < 0.001) and the play categories (Locomotor: *F*_(1,65)_ = 3.95, *p* = 0.051; Object: *F*_(1,67)_ = 5.12, *p* = 0.027; Social: *F*_(1,65)_ = 10.59, *p* = 0.002;). Birth weight was not associated with general activity (*F*_(1,52)_ = 0.14, *p* = 0.71). We also found BMI to be positively associated with total play and object play (e.g. total play: *F*_(1,65)_ = 4.95, *p* = 0.030); ponderal index was not associated with total play or the play categories. There was no statistical evidence that litter size at birth was associated with total play in this study.

**Fig. 4:**
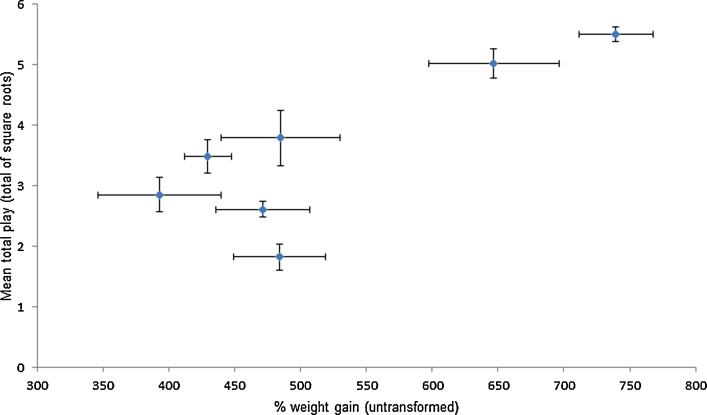
Graph of mean total play per litter (average total of square roots) against % weight gain (change in weight from birth to weaning) per litter. Horizontal error bars represent the SEM of the change in weight within the litter while vertical error bars represent the SEM of average total play counts within the litter.

Of the postnatal measures we found percentage piglet growth to be positively associated with total play (*F*_(1,67)_ = 10.02, *p* = 0.002; see Fig. 4) and the play categories (Locomotor: *F*_(1,67)_ = 3.98, *p* = 0.05; Object: *F*_(1,66)_ = 20.55, *p* < 0.001; Social: *F*_(1,67)_ = 7.83, *p* = 0.007).

When we sequentially added pre and postnatal measures to the fixed effects part of the model in a stepwise manner using REML we found variation across the play categories with respect to whether pre or postnatal measures entered the model first as the most highly significant term. Social play had a more highly significant association with birth weight than % weight gain to weaning whilst locomotor and object play showed the reverse. However, after adjusting for the first covariate, inclusion of the other covariate was still significant, indicating some association beyond that with the first covariate.

## 4 Discussion

The main aim of this paper was for the first time to analyse between and within litter differences in spontaneously occurring play behaviour in pre-weaned piglets. There is a general interest in individual behavioural differences and a growing awareness of their utility as an approach to the study of animal behaviour (e.g. Bell et al., 2009). However there are few studies that set out specifically to look for individual differences in play behaviour (Held and Spinka, 2011). For example in a study of play in Belding's ground squirrels (*Spermophilus beldingi*), Nunes et al. (2004) explored explanatory variables for spontaneous play in free-living squirrels but do not report directly on whether there were stable individual differences in play. Studies of dog ‘personality’ have suggested ‘playfulness’ as a stable personality trait, although these studies tend to use ‘tests’ of playfulness (e.g. Svartberg and Forkman, 2002) as opposed to observation of spontaneous play behaviour. For pre-weaned young in litter bearing species we need to take into account that variation in play may be affected by both individual and litter characteristics. Our study appears to be the first in any species to estimate the proportional distribution of variance in play between and within litters. In cats, previous work (Martin and Bateson, 1985) equalised litters and averaged play behaviour across the litter and found marked differences in play behaviour between litters. We have similarly identified litter differences in play. A recent study of play in wild dogs (*Canis familiaris*) did report within and between litter differences in play behaviour through the use of repeated Chi-square testing but was not able to comment on the relative strength of the these (Pal, 2010). Our REML analysis indicates that litter is a much stronger source of variation in play, over the six observation days that we used, than the individual piglet perhaps with the exception of object play. We also found variability in both litter and individual piglet play across different observation days.

Martin and Bateson (1985) pointed out that the causes and functions of the litter differences they observed in their cat study represented an important challenge for the study of behavioural development. In this study we can make some observations on potential explanatory factors for litter differences in play behaviour in pigs. We observed that the litter differences in play do not appear to be strongly related to litter differences in general activity. There was little evidence of between litter variation in general activity and the estimation of variance components for activity found a large residual variation which may indicate that play and activity are under the control of different causal factors. Similarly Martin and Bateson (1985) in their study of play in cats, found no evidence of litter differences in a measure of general activity. Furthermore, similar to Martin and Bateson (1985) we found that both the mean levels of total play and also the mean occurrence of different categories of play differed significantly between litters. Another possible explanation for the litter differences reported here is that in certain litters of pigs, play has a more ‘contagious effect’ with playing animals being more likely to stimulate play behaviour in other animals (e.g. Leca et al., 2007). We found that both the levels of what we defined as play invitations and rejections were strongly correlated with overall levels of play, and that there was no statistical evidence of the ratio of play invitations to rejections varying across litters. This would suggest that there was a similar proportional response to play invitations across litters and hence contagion is not having a strong influence on the litter differences in play we observed.

There have been only a few reported studies on the relationship between prenatal factors and development of play; for example Morley-Fletcher et al. (2003) reported that prenatal stress (caused by restraint of the mother) reduced social play in rats. In this study we found evidence that birth weight and to an extent BMI were associated positively with differences in total play and the play categories; ponderal index and litter size at birth were not associated with play. These relationships are partly explained by the correlations between these pre-natal variables (birth weight being correlated to BMI but not to ponderal index). Previous work in pigs (Litten et al., 2003) also reported a relationship between birth weight and play (measured in a standardised test) with low birth weight being associated with reduced play behaviour.

In terms of post-natal life we found a strong relationship between average litter levels of play and average litter growth between birth and weaning. Play is generally known to be sensitive to reductions in food availability with play generally declining along with food availability (e.g. deer (Müller-Schwarze et al., 1982); sheep (Reale and Bousses, 1999); meerkats (Sharpe et al., 2002) and primates (Baldwin and Baldwin, 1976)). Nunes et al. (2004) showed that body fat reserves were a constraint on expression of social play in ground squirrels under ecological conditions. As far as we can find there have been no studies which have associated variability of milk supply from a nursing mother and development of spontaneous play. Cameron et al. (2008) suggest that play behaviour in feral foals (*Equus caballus*) mirrors maternal investment (indicated by maternal condition). In domestic calves being artificially fed milk, play has been shown to be reduced by a low milk allowance (Duve et al., 2012). In a contradiction to the generally accepted relationship between nutrient availability and play, Bateson et al. (1981) found that interrupting lactation with bromocriptine led to an increase in levels of play in cats. In our study it seems most likely that the litter differences in growth rate relate to sow milk yield (e.g. Noblet and Etienne, 1989). There are however other possible explanations including across litter variation in the utilisation of milk nutrients by piglets (e.g. Aguinaga et al., 2011), or variation in levels of success with which piglets stimulated milk production from the sow (e.g. King et al., 1997, Farmer, 2013) or an interaction between these. Although litter size can influence growth in pigs (e.g. Auldist et al., 1998), in this study we found no association between litter size and play. Burghardt's (2005) surplus resource theory (SRT) proposes that play behaviour evolved where juveniles had available resources to use for play behaviour; hence play is most likely to evolve in young endotherms (with the ability to engage and recover from vigorous exercise), with extended juvenile phases with food and protection provided by parent(s). Generally our observation that postnatal growth and play are strongly associated appears to accord with the SRT although questions remain over the ‘rules’ that govern the allocation of resources between growth and play.

In general we found sex differences in play that agree with other studies. Males engaged in slightly more play overall as a result of them performing more non-harmful fighting behaviours and pushing behaviour than females. Proportionally females performed more locomotor play. Sexual dimorphism in play has been seen in other species (horses (*E. caballus*) Cameron et al., 2008; sheep (*Ovis aries*) Sachs and Harris, 1978) and it is suggested it plays a role in establishing social relationships with those likely to be interacted with in the future (Holmes, 1995). Male pigs would traditionally compete for access to females for mating (Graves, 1984), and the increased non-harmful fighting observed may support the ‘social training’ hypothesis of play development (Smith, 1982). Males also initiated more play events (with both male and female partners), supporting the hypothesis for a greater motivation for play initiation in males (e.g. Nunes et al., 2004). However, these sex differences cannot account for the total play difference between litters as sex ratios were reasonably consistent across litters within the population.

There is considerable interest in the longer-term consequences of play behaviour (e.g. Graham and Burghardt, 2010). Our study ceased at weaning. However it is worth noting that a recent study by Rauw (2013) on play in older (weaned) pigs, found that the litter of origin affected the number of play movements and time spent in play behaviour. This suggests that the litter effects we observed in our pre-weaning study may persist into the post-weaning phase of life.

In relation to animal welfare there has been increasing interest in the concept of ‘positive welfare’ (i.e. moving beyond providing for minimal welfare standards; e.g. Yeates and Main, 2008), and play behaviour has been proposed as a potential indicator for enhanced, positive welfare states (e.g. Lawrence, 1987, Held and Spinka, 2011). The results we present here support using play as an indicator of positive welfare in the pre-weaned pig. The litter differences in play we observed were associated positively with physical development (birth weight and weight change between birth and weaning). If play is to be used as an indicator of positive welfare in a practical setting then we will need to develop efficient approaches for measuring play. Previously Newberry et al. (1988) proposed the use of specific play elements as ‘play markers’. In this study the proportion of counts ‘run’ and ‘flop’ were positively correlated with total play suggesting these behavioural elements have the potential to be used as play markers in future studies of play in pre-weaned pigs. Future work should aim to examine why litters show differences in play behaviour, both in total play and elements of play, and what effect this may have on the piglets’ development.

## 5 Conclusions

As far as we are aware this is one of only a few studies that have set out to look for stable individual differences in play behaviour and the first time litter differences in play behaviour have been shown in pre-weaning pigs. The litter differences in play we observed, appear independent of activity levels, and were associated strongly with post-natal growth. We also found some evidence of pre-natal developmental effects on play and confirmed previously observed sex effects on the different categories of play. We conclude that the study of differences between litters and individuals provides a robust approach to understanding factors potentially influencing play behaviour in the pig. This work also provides support for the use of play as a welfare indicator in pre-weaned piglets as the litter differences in play we observed were associated positively with physical development.

## Figures and Tables

**Table 1:** Ethogram for piglet behaviours. Behaviours have been referenced to studies which have used the same or similar definitions. Invitation, and the play behaviour used to invite, were not mutually exclusive however neither invite nor reject counts were used in the analysis of total play and play categories.

Behaviour/group	Definition/type	References
**Locomotor play**		
Running	Energetic running and hopping in forward motions within the pen environment. Often associated with excitability, using large areas of the pen, and occasionally coming into marginal/accidental contact with other piglets (e.g. nudge)	Bolhuis et al. (2005), Chaloupkova et al. (2007), Donaldson et al. (2002), Newberry et al. (1988)
Pivot	Twirling of body on the horizontal plane by a minimum of 90° usually associated with jumping on the spot	Chaloupkova et al. (2007), Donaldson et al. (2002), Newberry et al. (1988)
Flop	Focal animal drops to the pen floor from a normal upright position to a sitting or lying position. There is no contact with an object or another individual (piglet or sow) which could cause the change of position	Chaloupkova et al. (2007), Donaldson et al. (2002)
Hop	Focal animal has either its two front feet or all four feet off the pen floor at one time through an energetic upwards jumping movement. The animal continues facing the same original direction for the whole of the behaviour	Newberry et al. (1988)

**Social play**		
Nudge	Snout of focal piglet is used to gently touch another piglet's body, not including naso-naso contact. Usually occurs in bouts of behaviour in quick succession. More intensive than mere touching, more gentle than a push	Donaldson et al. (2002)
Push	Focal animal drives its head, neck or shoulders with minimal or moderate force into another piglet's body. Occasionally the behaviour results in the displacement of the target piglet. Significantly more intensive than nudging	Blackshaw et al. (1997), Chaloupkova et al. (2007)
Climb	Placing both front hoofs on the back of another piglet or sow	Bolhuis et al. (2005)
Non-harmful fighting	Two piglets mutually push in a head to head orientation. A general mild intensity of the performed fighting behaviours distinguished non-harmful fighting from potentially harmful fighting	Defined for this study

**Object play**		
Object play	Animal manipulates an item or securely holds it in its mouth, energetically shaking it or carrying it around the pen	Newberry et al. (1988)

**Miscellaneous**		
Invite	Focal piglet performs play behaviours, which are clearly directed at another non-playing piglet. The behaviours are often repeated rapidly and are highly energetic	Martin et al. (2015)
Reject	Focal piglet which is a target of play invitation behaviours from another piglet, responds by turning its head and body away from the ‘inviting’ piglet and does not reciprocate any play behaviours or does not react to the inviting piglet's attempts at all.	Martin et al. (2015)

**Table 2:** The results of the REML analysis represented as contributions of each component (litter, litter × observation day, piglet within litter, residual) to variation in total play, the three play categories (locomotor, object and social play) and activity. The values in parentheses are the overall percentage contributions of the components to variance in play behaviour averaged over the six assessments for any randomly selected pig of any given sex.

	Litter	Litter × observation day	Piglet within litter	Residual	Total
Total play	1.181 (50%)	0.576 (24%)	0.270 (11%)	0.340 (14%)	2.37
Locomotor play	0.514 (41%)	0.384 (31%)	0.145 (12%)	0.210 (17%)	1.254
Object play	0.105 (23%)	0.118 (26%)	0.099 (22%)	0.136 (30%)	0.459
Social play	0.486 (50%)	0.172 (18%)	0.154 (16%)	0.167 (17%)	0.979
Activity	0.000 (0%)	0.045 (22%)	0.005 (2%)	0.160 (76%)	0.210

**Table 3:** The results of the REML analysis represented as contributions of each component (litter, litter × observation day, piglet within litter, residual) to variation in the different play elements. The values in parentheses are the overall percentage contributions of the components to variance in play elements averaged over the six observation days for any randomly selected pig of any given sex.

	Litter	Litter × observation day	Piglet within litter	Residual	Total
Nudge	0.085 (44%)	0.038 (19%)	0 (0%)	0.071 (37%)	0.194
Push	0.175 (47%)	0.047 (13%)	0.063 (17%)	0.086 (23%)	0.371
Non-harmful fighting	0.192 (41%)	0.085 (18%)	0.089 (19%)	0.108 (23%)	0.473
Flop	0.031 (30%)	0.014 (13%)	0.025 (24%)	0.035 (33%)	0.105
Hop	0.001 (6%)	0.001 (8%)	0 (0%)	0.010 (86%)	0.012
Pivot	0.011 (16%)	0.012 (17%)	0 (0%)	0.047 (67%)	0.070
Climb	0.042 (37%)	0.015 (13%)	0.016 (14%)	0.04 (36%)	0.112
Run	0.428 (39%)	0.356 (32%)	0.133 (12%)	0.179 (16%)	1.096
Shake	0.110 (25%)	0.106 (25%)	0.098 (23%)	0.119 (27%)	0.432
Carry	0.007 (9%)	0.009 (13%)	0.001 (2%)	0.055 (76%)	0.072
